# Supplementary material for: Controlling the first wave of the COVID–19 pandemic in Malawi: Results from a multi-round study
Source: PLOS Glob Public Health. 2024 Oct 24;4(10):e0003474. doi: 10.1371/journal.pgph.0003474 (PMC11500973; doi:10.1371/journal.pgph.0003474)
Supplement: S1 Appendix — (DOCX) [file pgph.0003474.s001.docx]

**S1 Appendix: GEOGRAPHIC DISTRIbUTION OF RESPONDENTS**

*Notes:* reported district of residence of study respondents during round 1. In our survey answer categories, Mzimba district was not split as Mzimba north vs. Mzimba south, as a result, we present data for these two districts jointly on the map.
