# Supplementary material for: Controlling the first wave of the COVID–19 pandemic in Malawi: Results from a multi-round study
Source: PLOS Glob Public Health. 2024 Oct 24;4(10):e0003474. doi: 10.1371/journal.pgph.0003474 (PMC11500973; doi:10.1371/journal.pgph.0003474)
Supplement: S2 Appendix — (DOCX) [file pgph.0003474.s002.docx]

**S2 Appendix: CHANGES IN HOUSEHOLD OCCUPANCY RATIO**

*Notes:* the y-axis is plotted on a logarithmic scale. The results presented in this graph are derived from two survey questions asking respondents 1) how many people reside in their household, and 2) how many rooms there are in their house. The occupancy ratio is then simply the number of household residents divided by the number of rooms. A log-linear regression model suggests household occupancy ratios declined over time in rural areas, but not in urban areas
