# Supplementary material for: Controlling the first wave of the COVID–19 pandemic in Malawi: Results from a multi-round study
Source: PLOS Glob Public Health. 2024 Oct 24;4(10):e0003474. doi: 10.1371/journal.pgph.0003474 (PMC11500973; doi:10.1371/journal.pgph.0003474)
Supplement: S3 Appendix — (DOCX) [file pgph.0003474.s003.docx]

**
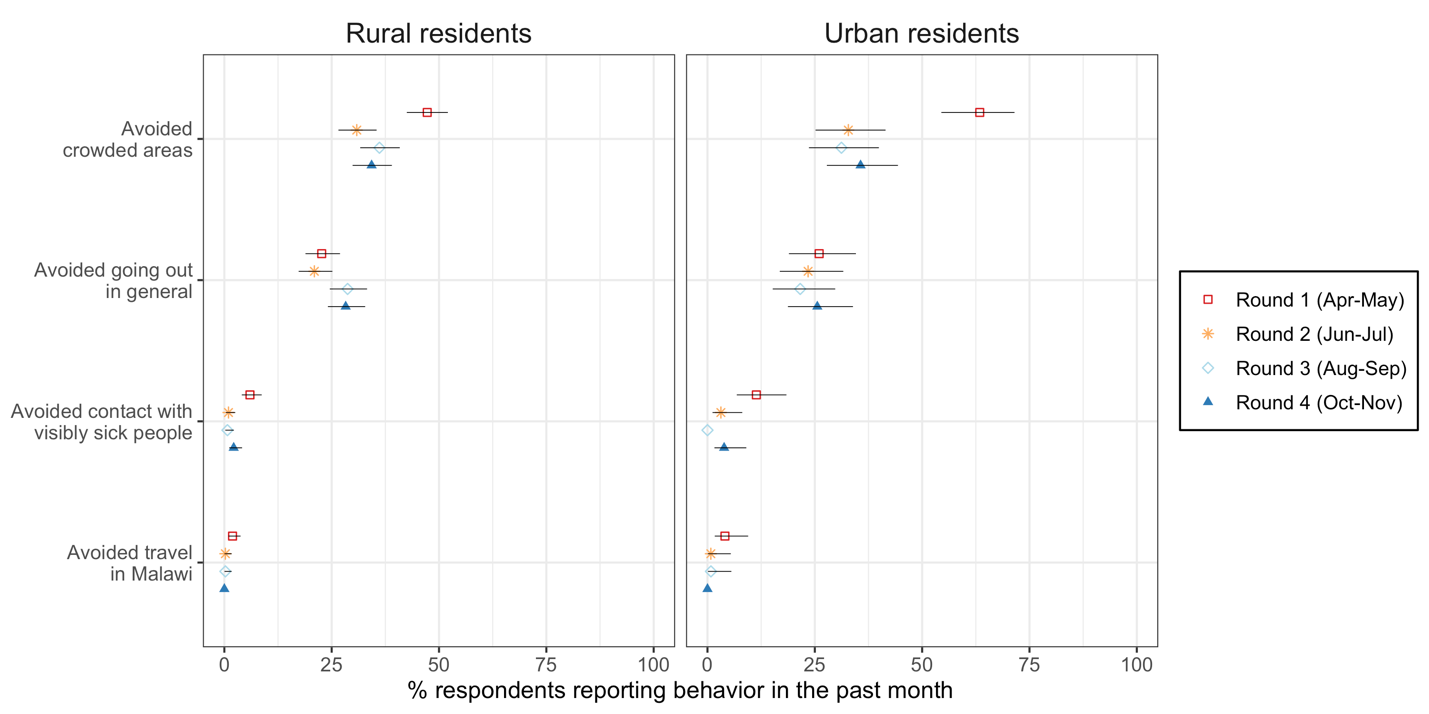
S3 Appendix: Adoption of behaviors to reduce contacts between infected and susceptible individuals in Malawi (April to November 2020)**

*Notes:* In this graph, the behaviors listed on the y-axis are those that reduce the rate of contact between infected and susceptible members of a population. In each round, <1% of panel respondents reported not doing anything to prevent the spread of SARS-CoV-2. The behaviors that appear on the y-axis are ordered according to their prevalence in urban areas in round 1. Error bars represent 95% confidence intervals.
