# Supplementary material for: Controlling the first wave of the COVID–19 pandemic in Malawi: Results from a multi-round study
Source: PLOS Glob Public Health. 2024 Oct 24;4(10):e0003474. doi: 10.1371/journal.pgph.0003474 (PMC11500973; doi:10.1371/journal.pgph.0003474)
Supplement: Attachment — Submitted filename: PLOS GPH - reviews.docx [file pgph.0003474.s006.docx]

Below, we present our point-by-point reply to the comments received from reviewers. The original comment appears in bold, and our reply appears in italic immediately below. When we cite revised text, we refer to the line numbers in our clean manuscript.

**Reviewer #1:**

**Is it possible to include a notion about governmental communication regarding the preventive behaviors studied? Where all measures pushed in a similar way in Malawi, or did you have politicians for example stressing the use of mouth masks and being an example already before they became compulsory?**

*We thank the reviewer for this question. We have added descriptions of the ways through which the MOH and other governmental/political parties communicated about the spread of the SARS-CoV-2 virus and the prevention of COVID-19. This included, for example, daily briefings about new case counts, as well as messages shared via social media platforms. However, we could not locate more systematic data on messages about COVID-19 prevention broadcasted through other sources, including politicians, media personalities, pastors and preachers and/or artists. Mask use was recommended well before it became mandatory in the country. This section now reads as follows:*

*“The Ministry of Health (MoH) and other stakeholders, initiated information campaigns (e.g., via radio messages, loudspeakers or SMS) to increase awareness and knowledge of the pandemic among the population. This included communications about how SARS-CoV-2 spreads, information about possible symptoms, and what to do when presenting such symptoms. The MoH established a toll-free hotline, as well as social media pages, where updates about the epidemic situation in Malawi, and recommendations, were posted. The MoH also conducted daily briefings about the epidemic situation, during which a spokesperson provided updated information about new cases of COVID-19 confirmed in the country, and periodically diffused messages about preventive behaviors.” (lines 339-348)*

**Reviewer #2:**

**The topics of this paper are interesting, though well known in other contexts. The structure and content must be revised, and results have to be better explained by authors before to be reconsidered for publication.**

- **Abstract has to be clarified the goal, provide empirical results, and health and social policy implications of results.**

*In response to this comment, we have revised the abstract to include the requested components.*

- **Introduction has to better clarify the research questions of this study and provide more theoretical background (See suggested readings that must be all read and used in the text). Hypothesis is too long; it has to be shortened in 2-3 lines.**

*We have edited the introduction, to better delineate the research questions. We have also shortened the hypothesis as requested. It now meets the requirement of brevity set by the reviewer, and it reads as follows:*

*“In the absence of a lockdown, individuals primarily respond to the health threat posed by COVID-19 by adopting behaviors that reduce the risk of transmission during contacts, rather than by limiting contacts.” (lines 123-125)*

- **Section 2 has to "theoretical background" as title.
  First, authors have to avoid a lot of subheadings that create fragmentation and confusion. If necessary, can use bullet points (same comments for all sections).**

*In response to this comment, we have named this section “Theoretical background”. We have also removed many of the subheadings, remaining with only 2 (“epidemiological framework”, “policy responses in African countries”). We have not used bullet points, as these do not seem to match the style of PLOS Global Public Health, but we hope that our revisions have clarified the presentation of this section (and subsequent sections as well). In responses to other comments formulated by the reviewer, and listed below, we will detail how we have improved the content of this section.*

- **Authors have to better describe the different sources of transmission dynamics of COVID-19 (e.g., climate, air pollution, density, etc.) and risk factors in society, which can accelerate diffusion of novel coronavirus in environment and relate nonpharmaceutical measures of control. After that they can focus on the topics of this study to provide a correct analysis for fruitful discussion (See suggested readings that must be all read and used in the text).**

*We have considerably added to the section on theoretical background, and in particular to the sub-section on “epidemiological framework”, to cover the factors described by the reviewer. We have thus implemented the following changes:*

1. *We have provided a description of COVID-19 disease and associated symptoms and risk factors (page 6, lines 152-161);*

2. *We have added a discussion of environmental factors on page 8, including air pollution, wind levels, temperature levels and humidity (lines 203-210);*

*;*

3. *We have provided a more thorough description of interventions against SARS-CoV-2, including contact tracing (p.9, lines 219-233);*
4. *finally, we have included a description of the role of population density in explaining contact patterns (p9, lines 212-217).*

*In making these revisions, we have extensively cited references provided by the reviewer (thank you for all these suggestions!)*

- **Methods of this study is not clear. The section of Materials and methods must be re-structured with following three sections only and same order to be clear for readers:
  • Sample and data
  • Measures of variables. They have to be clearly indicated and described
  • Models and Data analysis procedure.**

*As requested, we have simplified the presentation of this section, and we have adopted the subdivisions recommended by the reviewer. We have also strengthened our statistical methods, and we have revised the presentation of these methods in our main text. We believe that these revisions to the structure and content of this section clarify the presentation.*

- **Results.
  To reiterate, avoiding sub-headings that create fragmentation of the paper.
  Table 1 has to indicate in title that it is related to Malawi and the period under study.
  Pseudio-R2 is low this suggest a misspecification of the model. I suggest revising the measures used in modelling.**

*Here, we have removed sub-headings, as requested. The section on “results” no longer contains sub-headings. We have added “Malawi” and the period under study to the title of tables 1 and 2.*

*The reviewer also points out low R^2^ values in our statistical models of the adoption of masking and physical distancing. We have carefully considered this remark. In response, however, we did not revise the measures/variables used in our statistical modeling of behavioral outcomes for two reasons. On the one hand, our covariates include standard sociodemographic characteristics of respondents, e.g., their age or educational level. Since our interest is in describing the characteristics of early adopters of these strategies, we considered that it was important to maintain these covariates. On the other hand, our panel data was collected by mobile phone. Due to this mode of data collection, it only included limited additional data on the socioeconomic characteristics of respondents (to maintain interviews short). We thus cannot add variables to our statistical models to try and improve our ability to predict behavioral outcomes.*

*To discuss the limited predictive power of our statistical models, we thus chose to include this aspect as a limitation in our discussion section. This added component reads as follows:*

*“Fifth, even though we used a flexible specification, our multivariate models either failed to identify covariates of the adoption of preventive behaviors, or only accounted for a small proportion of the variability in practices across panel participants and over time. This might have been the case because we focused on a small set of independent variables, which described key sociodemographic characteristics of respondents. As in studies of attitudes and behaviors related to other diseases (e.g., HIV), more detailed investigations of the adoption of preventive behaviors related to COVID-19 could explore factors related, for example, to an individual’s social networks or his sources of information (126,127). Unfortunately, we did not collect such data in our panel study, in large part due to the need to keep interview duration short in surveys conducted by mobile phone.” (lines 819-829)*

- **Figure 2. Data have to be normalized with population on y-axis to be reliable. Analysis it should be better that is done also considering the third wave.**

*As requested, we have stretched the time axis of the plot, to include subsequent waves of the COVID-19 pandemic in Malawi. We describe the occurrence of these subsequent waves in the main text. We clearly state that our panel data on behavioral outcomes stops in November 2020, and thus does not cover the periods that include the 2^nd^ and 3^rd^ waves of the COVID-19 pandemic in the pandemic. This added passage reads as follows:*

*“Subsequently, Malawi experienced additional waves of the pandemic, which were linked to new variants of the SARS-CoV-2 virus. These occurred in early 2021, with peaks at more than 1,300 recorded confirmed cases in a day, and in July and August 2021, with peaks at approximately 1,000 recorded confirmed cases in a day. Our longitudinal data however do not cover these periods (figure 2).” (lines 344-348)*

*We have not reported rates per 1,000 people on the y-axis, as suggested by the reviewer. We have decided not to do so because a) our figure focuses on the population of a single country (Malawi), and b) there are no data in Malawi on how population size changes from month to month. Since it focuses on a single country, reporting raw numbers of cases is not subject to confounding due to issues of scale (which would emerge if we were comparing Malawi to a much larger country like, say, Nigeria or Ethiopia). In addition, issues of data availability about population size preclude this adjustment. This is the case because Malawi has not set up a population register that allows monitoring population changes on a finer time scale, as for example in Scandinavian countries. Instead, a full count of the population is only available for 2018, i.e., the year of the last population census. Given that the population of Malawi is growing at a relatively rapid rate, it would be misleading to scale the reported cases by population size in 2018. This might bias estimated incidence and mortality rates upwards (due to a denominator that would be too low), and the magnitude of this bias would grow over time. After 2018, there are only population projections that are available, but those make a number of assumptions that might not be plausible at the time of a global pandemic (e.g., regarding migration patterns or the age pattern of mortality). Finally, please note that we provide the size of the population of Malawi in our text, so that readers can calculate (approximate) rates of COVID-19 cases per capita if needed.*

- **Figure 3. avoid in legend acronyms and use full words. The arrows are not clear what indicate. In any case this figure is not clear…and messy.**
- **Figure 4 and 5-6 same comments for figure 3.**

*Thank you for this comment. We have avoided all acronyms in the legends of our figures. We have also significantly revised these figures for clarity. We have removed the arrows, which introduced confusion, as noted by the reviewer. We have included confidence intervals around each of the estimated proportions, so that the reader can benefit from measures of uncertainty. We have also removed the different levels of transparency that might have introduced confusion in prior versions, as well as difficulties in reading the plots. We believe that the revised figures greatly improve the clarity of our presentation.*

- **Analysis are related to 2020, and they have to better used to show how can be used to face next pandemics of new viral agents, when drugs are missing, in particular in terms of timing of mask wearing and behavioral change.**

*Thank you for this comment. We cover our answer to this point further below, as it relates primarily to our discussion of study findings.*

- **The paper has a lot of figures that are difficult to digest, some of them can be put in appendix and inserting in the text the most important ones to improve the readability…**

*Following this point, we have a) revised our figures to simplify them and make them more legible, as detailed above, and b) moved some figures to the appendix as suggested. Our paper now has 2 tables and 5 figures, while 4 tables and figures are retained for the appendix. We hope this addresses the reviewer’s concern.*

- **As there is a comparison between rural and urban residents, a reliable comparative analysis should consider the differences of values to show the dynamics and design a strategy of intervention.
  Clarify urban residents to which cities they belong.**

*In response to this point, we have provided a detailed description of the respondents classified as urban in our analyses. It appears on page 20 and reads as follows:*

*“We used the definition of “urban” areas from the Malawi National Statistical Office: “urban centers in Malawi refer to the four major cities [of] Blantyre, Lilongwe, Mzuzu, and Zomba, town councils and bomas (i.e., the administrative capital of each district) and other town-planning areas” (103). All other areas of residence were classified as “rural”. (lines 468-472)*

- **Discussion.
  First, authors have to synthesize the main results in a simple table to be clear for readers and then show what this study adds compared to other studies. Authors should provide with bullet points, clear best practices of health policy to cope with pandemic diffusion in rural and urban residents.**

*As recommended by the reviewer, our discussion starts with synthesis of the main results of the paper. We also discuss how our findings relate to the findings from previous studies. In doing so, we focus particularly on studies which were conducted in other African settings, particularly those where lockdowns or stay-at-home orders were imposed. We chose not present our results in terms of “best practices” however, because we do not believe that our results would be sufficient to formulate such recommendations. Indeed, best practices are usually obtained through a review multiple studies, which are then summarized, evaluated, and possibly pooled and analyzed jointly. The formulation of “best practices” in global health also often entails consultation of experts. Our study, on the other hand, describes the experience of a sample of individuals in the context of one specific country (Malawi). It might be included in future systematic reviews of the evidence on behavioral change during the COVID-19 pandemic, but by itself, we do not believe that it warrants labeling our findings as “best practices”.*

- **Conclusion has to be an autonomous section. Conclusion has not to be a summary, but authors have to focus on manifold limitations of this study and provide clear implications of health policy, as well as how nations can prevent and mange with good governance and new technologies (mechanical ventilation) next pandemics with vaccination and nonpharmaceutical measures of control.**

*We have added a conclusion to our paper. We kept this section short, as appears to be the norm in PLOS Global Public Health. We did not reiterate the limitations of our study, as those are presented in much detail in the earlier “discussion” section. The added conclusion reads as follows:*

*“Our study has described how individuals navigated the new health threat posed by COVID-19 in Malawi, a country where no requirements to stay at home were imposed during the first months of the pandemic. In that context, study participants reported wearing masks and practicing physical distancing as their main responses to the spread of SARS-CoV-2. The implementation of these strategies coincided with an epidemic downturn, suggesting that behaviors that reduce the transmissibility of SARS-CoV-2 during contacts might have played a key role in controlling the first wave of the COVID-19 pandemic in Malawi. The adoption of mask use and physical distancing was however uneven in our sample, with members of various disadvantaged socioeconomic groups reporting a delayed adoption of these protective practices. Our findings thus stress a) the importance of fostering protective behavioral changes to contain future outbreaks of SARS-CoV-2 in settings where access to vaccination remains low, and b) the need for targeted outreach to members of socioeconomic groups in which the adoption of protective behaviors might be delayed.” (lines 856-869)*

- **Overall, then, the paper is interesting, but theoretical framework is weak, and some results are vague and create confusion… structure of the paper has to be improved; study design, discussion and presentation of results have to be clarified using suggested comments. To be clear, I strongly suggest improving the paper, by using all comments (suggested papers included to read and use all) that I will in-depth verify, and maybe it can be considered. If the paper is not improved as suggested it will be dismissed.**

*Thank you once again for the careful review of our work. As mentioned in our responses to the points above, we have taken all comments into account and we have revised our paper extensively. We have also carefully reviewed the suggested references, and we have cited them in the revised version of our manuscript.*

**Reviewer #3: This well-written paper is potentially useful to guide other researchers and practitioners in other COVID-19 studies. Saying that, I have a few comments, meaning to improve the discussion.**

- **The findings confirm previous studies indicating the efficacy of mask-wearing, even in settings of poor social distancing in reducing COVID-19 transmission (https://www.nature.com/articles/s41467-021-24115-7). However, some caution is needed to interpret the results.**

*We thank the reviewer for this reference, which we now cite in our discussion.*

- **(a) Malawi´s age structure indicates that only 2.68% of the population was 65+ yo in 2020, making the elderly, a more vulnerable population, relatively small;
  (b) The urban population accounts for only 18% of the total population - and COVID-19 transmission is much reduced in rural settings due to the sparser population.
  So, it is no surprise that the number of severe cases and deaths was less than initially expected. In this situation, it should be interesting to discuss these two factors in this context.**

*Thank you for this comment. We have added discussion of the youthful age structure and rate of urbanization in our description of the context. The projections of potential impact formulated in early 2020 accounted for these parameters. We now describe the methodology of these projections more precisely. The added passages that address this comment read as follows:*

*“The population of Malawi is very young, with a median age of 17 years in 2018, and only 4 percent of the total population aged 65 years and older (103). It is also growing rapidly, at an annual rate of approximately 3 percent per year. Prior to the COVID-19 pandemic, Malawi had made significant progress towards the achievement of health goals related to HIV treatment (105,106) and to the reduction of child mortality (107). In recent years, the incidence of non-communicable diseases (e.g., neoplasms) has increased in urban and rural areas (108).” (lines 315-321)*

*“Early in the pandemic, it was projected that 1 in 5 Malawians might become infected with SARS-CoV-2 in 2020, resulting in more than 60,000 hospitalizations and close to 2,000 deaths (109). These projections accounted for some of the key factors that might affect the transmission of SARS-CoV-2 in Malawi relative to other African countries, including the large proportion of the population residing in rural areas, and expected levels of rainfall. As of the end of December 2020 however, Malawi had recorded 6,583 cases of COVID–19, resulting in 189 deaths.” (lines 336-342)*

- **Figure 1: We should view those map indicators with a grain of salt. The fact that the central governments issued a decree regarding stay-at-home orders does not mean that those orders were effectively applied. This is especially important in countries where the presence of the state is precarious and lacks the structure to enforce the lockdowns and provide adequate health care.**

*We agree with this comment, and we have responded to it in two ways: first, we have used the Oxford data in a more detailed manner, to show that the requirements to stay at home were imposed with different levels of exception across the continent; second, we mention different levels of implementation across African countries in our sub-section on limitations. As a result, our map in figure 1 is more nuanced than in our original submission, as it includes a more detailed classification of countries. We have also added the following paragraph about implementation and enforcement:*

*“Among the 50 African countries documented by the Oxford COVID-19 Government response tracker (9), 40 countries required (at least some of) their citizens to stay at home at some point during the period stretching from the start of the pandemic to November 1^st^, 2020. Some of these countries (e.g., Zambia, South Africa, Ghana) granted exemptions from lockdown measures for “essential” services and functions. Other countries (e.g., Botswana, Kenya) adopted stricter measures with only limited exceptions. The enforcement of lockdown measures also varied between countries (74–76), with instances of non-compliance with stay-at-home orders, and possibly hostility towards authorities, documented in multiple settings (77,78).” (lines 252-261)*

- **Figure 6: According to the graphs, rural and urban residents show nearly identical behavior regarding mask use, contrary to what was stated in some parts of the text.**

*We thank the reviewer for this remark. The figure that the reviewer refers to (which is now figure 5) pertains to the consistency of mask use among users in the latter rounds of our panel study. In that regard, there are indeed limited differences between urban and rural residents. On the other hand, there are larger differences between rural and urban residents in the scale and timing of the adoption of mask use, as a protective practice. This is shown on figure 4, and in table 2.*

- **It should be interesting to discuss the paper's findings in light of the following statement: "Closing all educational institutions, limiting gatherings to 10 people or less, and closing face-to-face businesses each reduced transmission considerably. The additional effect of stay-at-home orders was comparatively small", extracted from the article**
  [**https://pubmed.ncbi.nlm.nih.gov/33323424/**](https://pubmed.ncbi.nlm.nih.gov/33323424/)

*We thank the reviewer for this suggestion. However, this paper tackles a slightly different question than our own analysis. Specifically, it seeks to evaluate the effectiveness of various interventions (NPI) that reduce contacts within the population. In doing so, it does not consider mask wearing and physical distancing. The authors cite this aspect as a limitation of their analyses, which might confound their assessment of the relative effectiveness of various interventions to reduce contact. In our paper, we thus decided to focus on references in which interventions to reduce contact, and interventions to reduce transmission during contacts, are jointly considered.*
