# Supplementary material for: Controlling the first wave of the COVID–19 pandemic in Malawi: Results from a multi-round study
Source: PLOS Glob Public Health. 2024 Oct 24;4(10):e0003474. doi: 10.1371/journal.pgph.0003474 (PMC11500973; doi:10.1371/journal.pgph.0003474)
Supplement: Attachment — Submitted filename: Reply to reviewers.docx [file pgph.0003474.s007.docx]

Reply to reviewers

*We received the following comment in review of our revised manuscript:*

**Just a minor comment, which could be left at the discretion of the authors:
1) Please fix Figure 1: the Hala'ib Triangle region on the Egypt-Sudan border is missing from the map.**

*We thank the reviewer for noticing the issue with our map. We have fixed the figure to include this region. We have revised the caption of this figure, to indicate the source of our base map. We thus note that have obtained the new base map from the the World Bank repository of geographic information available at:* [*https://datacatalog.worldbank.org/search/dataset/0038272/World-Bank-Official-Boundaries*](https://datacatalog.worldbank.org/search/dataset/0038272/World-Bank-Official-Boundaries)

*This minor addition constitutes the only revision that we have brought to our manuscript.*
